# Serum Dioxin Concentrations and Age at Menarche

**DOI:** 10.1289/ehp.7004

**Published:** 2004-06-10

**Authors:** Marcella Warner, Steven Samuels, Paolo Mocarelli, Pier Mario Gerthoux, Larry Needham, Donald G. Patterson, Brenda Eskenazi

**Affiliations:** ^1^School of Public Health, University of California at Berkeley, Berkeley, California, USA; ^2^Division of Occupational/Environmental Medicine and Epidemiology, University of California at Davis, Davis, California, USA; ^3^Department of Laboratory Medicine, University of Milano-Bicocca, School of Medicine, Hospital of Desio, Desio-Milano, Italy; ^4^Division of Environmental Health Laboratory Science, National Center for Environmental Health, Centers for Disease Control and Prevention, Atlanta, Georgia, USA

**Keywords:** dioxin, endocrine disruptors, environmental exposures, epidemiology, menarche, puberty, 2, 3, 7, 8-tetrachlorodibenzo-*p*-dioxin

## Abstract

2,3,7,8-Tetrachlorodibenzo-*p*-dioxin (TCDD), a widespread environmental contaminant, is associated with delays in pubertal development in animal studies. On 10 July 1976, as a result of a chemical explosion, residents of Seveso, Italy, experienced the highest levels of TCDD exposure experienced by a human population. Twenty years later, we initiated the Seveso Women’s Health Study (SWHS), a retrospective cohort study of female residents of the most contaminated areas, to determine whether the women were at higher risk for reproductive disease. We examined the association of TCDD serum levels, based on measurements in serum collected soon after the explosion, with reported age at menarche among the 282 SWHS women who were premenarcheal at the time of the explosion. We found no change in risk of onset of menarche with a 10-fold increase in TCDD (e.g., 10–100 ppt; hazard ratio = 0.95; 95% confidence interval, 0.83–1.09; *p*-value for trend = 0.46). When TCDD levels were categorized, there was also no evidence of a dose–response trend (*p* = 0.65). In summary, we found that individual serum TCDD measurements are not significantly related to age at menarche among women in the SWHS cohort. The women in this study experienced substantial TCDD exposure during the postnatal but prepubertal developmental period. Given that animal evidence suggests *in utero* exposure has the most significant effect on onset of puberty, continued follow-up of the offspring of the SWHS cohort is important.

Polychlorinated dibenzodioxins (PCDDs), polychlorinated dibenzofurans (PCDFs), and polychlorinated biphenyls (PCBs) constitute a group of polyhalogenated aromatic hydrocarbons that are persistent, widespread environmental contaminants, frequently detected at parts-per-trillion levels (lipid basis) in animals and humans throughout the industrialized world (Zook and Rappe 1994). 2,3,7,8-Tetrachlorodibenzo-*p*-dioxin (TCDD or dioxin) is the most toxic congener within this group of compounds and has been shown to cause a wide variety of effects in animals, including altered reproductive development [Birnbaum 1994, 1995; International Agency for Research on Cancer (IARC) 1997]. Increasing evidence suggests that exposure to TCDD during earlier stages of development is particularly hazardous to reproductive development (Chaffin et al. 1996). *In utero* and lactational TCDD exposure in rodents has been associated with delays in pubertal development [e.g., delayed vaginal opening, altered vaginal estrous cyclicity (Gray and Ostby 1995; Wolf et al. 1999)] and effects on ovarian function (Gray and Ostby 1995; Heimler et al. 1998), even at doses below those that induce overt maternal toxicity. A similar spectrum of reproductive alterations has been associated in rodents exposed *in utero* to other dioxin-like compounds, including PCDDs, PCDFs, and PCBs (Faqi et al. 1998; Hamm et al. 2003; Muto et al. 2003; Sager and Girard 1994).

To date, no epidemiologic studies have examined the association of TCDD exposure and age at menarche. Three studies, however, have examined the relation of dioxin-like compounds to pubertal development, with inconsistent conclusions. A study of daughters of Michigan women who had consumed polybrominated biphenyls (PBBs) in food in 1973 found earlier menarche in daughters whose mothers had higher serum PBB levels (Blanck et al. 2000). There were no differences in age at menarche in Taiwanese women who were exposed postnatally (but premenarche) to PCBs and PCDFs via consumption of contaminated rice oil (Yucheng) compared with unexposed women (Guo and Kao 2003). In Flemish adolescents, although breast development was inversely related, there was no relation of age at menarche to current serum levels of dioxin-like compounds as measured by chemical-activated luciferase gene expression bioassay toxic equivalents (CALUX-TEQ) or individual PCB congeners 118, 153, and 180 (Den Hond et al. 2002).

On 10 July 1976, as a result of a chemical explosion, residents of Seveso, Italy, experienced the highest levels of TCDD exposure in a human population. Shortly after the explosion, a cohort of residents was established and exposure status was classified by zone of residence (A, B, R, non-ABR) as determined by surface soil TCDD measurements (di Domenico et al. 1980). Twenty years after the explosion, we initiated the Seveso Women’s Health Study (SWHS) to measure TCDD in previously stored blood samples and to assess associations of serum levels of TCDD with reproductive disease.

In the SWHS, we have observed that serum TCDD levels were associated with an increase in menstrual cycle length among those who were premenarcheal at exposure, but not in those who were postmenarcheal at exposure (Eskenazi et al. 2002). Consistent with animal studies (Chaffin et al. 1996), this suggests that females may be particularly susceptible to the effects of TCDD during early stages of development, for example, *in utero* or prepubertal exposure. In this article we report the results of the association of individual serum TCDD and age of menarche among women who were premenarcheal in 1976, at the time of the explosion.

## Materials and Methods

### Study population.

Women eligible for the SWHS were 1 month to 40 years of age in 1976, had resided in one of the most highly contaminated zones (A or B), and had adequate stored sera collected soon after the explosion. Enrollment began in March 1996 and ended in July 1998. Of 1,271 eligible women, 17 could not be located or contacted, 33 had died or were too ill to participate, and 240 declined to participate, leaving 981 women. The age distribution of those who declined to participate was not significantly different from those who did participate. For this analysis, we included all women who were premenarcheal on 10 July 1976, the date of the explosion (*n* = 282).

### Procedure.

The institutional review boards of the participating institutions approved the study. Details of the study have been presented elsewhere (Eskenazi et al. 2000). Briefly, participation included signed informed consent, blood draw, personal interview, and for most women, a gynecologic examination and ultrasound. The interview was conducted by a trained nurse-interviewer who was blinded to serum TCDD levels and zone of residence. Age at menarche was determined from the question, “At what age did you get your first menstrual period?”

### Laboratory analyses.

TCDD was measured in archived sera by high-resolution gas chromatography/high-resolution mass spectrometry methods (Patterson et al. 1987). Values are reported on a lipid-weight basis in parts per trillion (Akins et al. 1989).

Details of serum sample selection have been presented elsewhere (Eskenazi et al. 2000). For the 282 women in this analysis, we measured TCDD in sera collected between 1976 and 1977 for 257 women, between 1978 and 1981 for 23 women, and in 1996 for two women whose earlier samples had insufficient volume. For women with detectable post-1977 TCDD measurements (*n* = 20), the TCDD exposure level was back-extrapolated to 1976 using the Filser model (Kreuzer et al. 1997). For nondetectable values (*n* = 22), a serum TCDD level equal to one-half the detection limit was assigned (Hornung and Reed 1990). For the median serum sample weight of 0.65 g, the median detection limit was 18.8 ppt, lipid adjusted.

### Statistical analyses.

We considered serum TCDD both as a continuous variable (log_10_ TCDD) and a categorical variable. TCDD was first categorized into quartile groups (≤ 55.9, 56–140.2, 140.3–300, > 300 ppt). Because the lower limit of the serum TCDD level was relatively high, the lowest group was subdivided into women with levels ≤ 20.0 and 20.1–55.9 ppt. We selected 20 ppt (body burden ~ 4 ng/kg) as the cutoff point because this was the average TCDD level of 1976 serum pools collected from Italian women living in an unexposed area (Eskenazi et al. 2004). For additional analyses, we categorized preexplosion experience as “unexposed.”

Statistical analyses were performed using Cox survival models in Stata 7 (Stata Corporation, College Station, TX, USA). We did not censor data because for each woman age of menarche was observed. Each woman was entered into the denominator (“risk set”) for her year-group at the date of the accident or on her seventh birthday, whichever was later.

The Cox model assesses effects on age-specific probabilities of beginning menstruation by the relative hazard, or hazard ratio (HR), the ratio of probabilities computed for each categorized level of exposure versus the reference group or for the effect of a 10-fold increase in TCDD (log_10_ TCDD). For the categorical analysis, we used the highest dose group (> 300 ppt) as the reference group because the lowest dose group (≤ 20 ppt) had the smallest sample size. We report model-free standard errors, which are valid even when conventional assumptions for regressions are violated (Huber 1967). We examined the effect of potential confounders and effect modifiers, including height, weight, body mass index (BMI), and report of participation in athletic training at the time of interview (we did not obtain this information for early time periods), and smoking and alcohol consumption habits between 10 and 14 years of age.

The Cox model with constant HR may not be plausible when there is an inevitable event and the age-specific rates increase to 100%. We therefore also considered parametric regression survival-time models in which the natural log of the age at menarche is expressed as a linear function of the covariates.

The youngest age at menarche reported by the women who were premenarcheal at the time of the explosion was 8 years. We addressed the possibility of bias associated with the inclusion of women who, relative to their birth cohort, might already be at risk for late age at menarche at the time of the explosion (e.g., a woman who was 14 years of age but still premenarcheal in 1976). We therefore repeated the analysis on the subset of 158 women who were < 8 years of age at the time of the explosion and who were presumably not yet at risk for menarche.

To further assess the possibility of bias, we added to the analysis data 153 women who were in the same birth cohort as the 282 women in the analysis sample (birth years 1959–1976) but who had begun menstruating before the explosion date, 10 July 1976. These 153 women would have been at risk for menarche after the explosion had they not reached menarche before the explosion; their premenarche ages are all “unexposed.” For the enlarged sample of 435 (153 + 282) women, we repeated the analysis with this additional “unexposed” exposure category (unexposed, ≤ 20, 20.1–55.9, 56.0–140.2, 140.3–300, and > 300 ppt) and each “unexposed” woman entered the denominator on her seventh birthday.

## Results

Demographic characteristics of the 282 women who were premenarcheal at exposure are presented in Table 1. On 10 July 1976, the age of the 282 women was 6.9 ± 3.7 years (mean ± SD; range = 0–17 years), and 158 (56%) were < 8 years of age. The mean age at menarche reported for the 282 women was 12.8 ± 1.6 year. The mean age at follow-up (1996–1998) was 27.3 ± 3.8 years and the mean BMI was 21.4 ± 3.1 mg/kg^2^. Women who had higher current BMIs or who consumed alcohol or smoked regularly between 10 and 14 years of age reported earlier ages of menarche.

Serum TCDD levels are presented in Table 2 by reported age at menarche for all premenarcheal women (*n* = 282) and for women who were < 8 years of age (*n* = 158) at exposure. The median serum TCDD level was 140.3 ppt (range, 3.6–56,000 ppt) for all premenarcheal women and 205.0 ppt (range, 3.6–56,000 ppt) for those who were < 8 years of age at exposure. Serum TCDD levels did not vary by reported age of menarche for either group [*p* > 0.5, analysis of variance (ANOVA)].

Results of Cox models are presented in Table 3. When we examined the effect of potential confounders and effect modifiers, we found no variables to confound (i.e., change the TCDD parameter estimate by > 10%) or to modify the association between TCDD and age of menarche. Thus, we report unadjusted results. When log_10_ TCDD was entered as the exposure variable, the HR associated with a 10-fold increase in TCDD was 0.95 [95% confidence interval (CI), 0.83–1.09]. That is, there was no change in risk of onset of menarche with a 10-fold increase in TCDD levels (e.g., 10–100 ppt). When the analysis was restricted to women < 8 years of age at exposure, the HR associated with a 10-fold increase in TCDD was 1.08 (95% CI, 0.89–1.30).

When TCDD was categorized (Table 3), there was also no evidence of a dose–response trend (*p* = 0.65). None of the four lower exposure groups (≤ 20.0, 20.1–55.9, 56–140.2, 140.3–300 ppt) had significantly different age-specific menarche rates than the highest category (> 300 ppt), and all CIs for the HR contained 1.0. The conclusions were similar when the analysis was restricted to women who were < 8 years of age at the time of the explosion. The conclusion of no association of age at menarche and TCDD persisted when we applied alternative models (log-normal, log-logistic) in which the mean of the log of age at menarche was the response (results not shown).

Finally, the analysis of all 435 women in the 1959–1976 birth cohort, with preexplosion ages categorized as “unexposed” also showed no association of TCDD level and age-specific hazard of menarche (data not shown).

## Discussion

The results of this study of women residing in Seveso, Italy, in 1976 at the time of an explosion that released high levels of TCDD provide little evidence of an association of exposure and age of menarche. That is, we found no evidence of an association between TCDD levels measured in serum collected near the time of exposure among the 282 women who were premenarcheal at the time of the explosion, the subset of 158 women who were < 8 years of age at the time of the explosion, or the 435 women who belonged to the 1959–1976 birth cohort.

A limitation of our study is the retrospective recall of age of menarche. However, moderate to high correlations between actual and recalled menarche have been reported for females up to 19 years of age after the event (Must et al. 2002). In our study, the time between onset of menarche and study interview ranged from 5 to 19 years. The women in the SWHS reported age at menarche in whole years, presumably age at last birthday, and age was not rounded to the nearest “biological age.” Such nondifferential measurement error would reduce precision and would tend to bias our findings toward no effect.

A second limitation of the present study is that members of the lowest TCDD exposure group (≤ 20 ppt) experienced relatively high serum levels in comparison with contemporary levels reported for this area (~ 2 ppt) (Warner M, unpublished data). If there is a threshold for TCDD effects on age of onset of menarche but it is < 20 ppt, we would not be able to detect it in this population. However, we also found no association in analyses that counted preexplosion experience as “unexposed.”

Another limitation of this study is that, although the explosion resulted in exposure specifically to TCDD, analyses of pooled serum from residents of an unexposed zone suggest there was substantial background exposure to other PCDDs, PCDFs, and PCBs during this time period [80 ppt TCDD toxic equivalents (TEQ), on average] (Eskenazi et al. 2004). Therefore, individuals with TCDD levels < 20 ppt might still have had substantial total TEQ exposure. Because we considered only TCDD in this study, our results may have underestimated an effect due to total TEQ exposure.

An advantage of this study over previous studies is that we were able to measure TCDD levels in individual serum samples collected near the time of exposure. Previous studies have used cross-sectional exposure measures (Den Hond et al. 2002) or had to rely upon alternative exposure assessment methods including ecologic measures (Guo and Kao 2003) or modeling (Blanck et al. 2000).

Our finding of no association of TCDD with age at menarche is consistent with results reported in studies with postnatal exposure to other dioxin-like compounds, including PCBs and PCDFs (Den Hond et al. 2002; Guo and Kao 2003). However, our results differ from those of Blanck et al. (2000), which, in contrast to animal studies, showed an earlier rather than later age of menarche with *in utero* and perinatal PBB exposure. There are several reasons why our results may differ. The PBB studied by Blanck et al. (2000), 2,2′,4,4′,5,5′-hexabromobiphenyl, which was the main congener (60–80%) in the Fire Master mixture, is not a dioxin-like congener and does not bind to the aryl hydrocarbon receptor, unlike coplanar PBBs (Darnerud 2003). Second, the PBB-exposed cohort was exposed *in utero* and via lactation, unlike the SWHS cohort, in whom no exposure occurred *in utero* and only three women reported having been breast-fed postexplosion. It is possible that the fetus is more sensitive to the effects of exposure to dioxin-like compounds *in utero*. In fact, although *in utero* and lactational TCDD exposure in animal studies has been associated with significant effects on onset of puberty (Gray and Ostby 1995; Wolf et al. 1999) and ovarian function (Gray and Ostby 1995; Heimler et al. 1998), the evidence for these adverse effects after only postnatal exposure is limited, based on studies using the immature intact and immature hypophysectomized rat models (Gao et al. 1999; Li et al. 1995, 1997; Son et al. 1999). Thus, postnatal (but prepubertal) TCDD exposure experienced by the SWHS cohort, although substantial in dose, likely missed the critical window for exposure effects.

In summary, we have shown that individual serum TCDD measurements are not significantly related to age at menarche among women in the SWHS cohort. The women in this study experienced substantial TCDD exposure during the postnatal but prepubertal developmental period. Given that animal evidence suggests *in utero* exposure has the most significant effect on onset of puberty, continued follow-up of the offspring of the SWHS cohort is important.

## Figures and Tables

**Table 1:** Distribution and age of menarche by select characteristics of women who were premenarcheal at exposure, SWHS, Seveso, Italy, 1996–1998.

			Age at menarche (years)	
Characteristic	All premenarcheal women [No. (%)]	Women < 8 years of age in 1976 [No. (%)]	All premenarcheal women (mean ± SD)	Women < 8 years of age in 1976 (mean ± SD)
Total	282 (100)	158 (56)	12.8 ± 1.6	12.5 ± 1.5
Age at exposure (years)				
0–4	84 (30)	84 (53)	12.6 ± 1.5	12.6 ± 1.5
5–7	74 (26)	74 (47)	12.4 ± 1.6	12.4 ± 1.6
8–10	69 (24)	0 (0)	12.4 ± 1.3	—
11–17	55 (20)	0 (0)	13.8 ± 1.5	—
Year of birth				
1959–1966	93 (33)	0 (0)	13.3 ± 1.5	—
1967–1970	90 (32)	59 (37)	12.4 ± 1.6	12.6 ± 1.7
1971–1976	99 (35)	99 (63)	12.5 ± 1.4	12.5 ± 1.4
Zone of residence				
A	58 (21)	37 (23)	13.0 ± 1.8	12.6 ± 1.2
B	224 (79)	121 (77)	12.7 ± 1.5	12.5 ± 1.6
Current BMI (kg/m^2^)				
< 19.8	93 (33)	63 (40)	13.2 ± 1.6	13.1 ± 1.6
19.8–26	166 (60)	87 (55)	12.6 ± 1.5	12.2 ± 1.4
26–29	12 (4)	5 (3)	12.5 ± 2.5	11.4 ± 1.9
> 29	8 (3)	2 (1)	12.4 ± 1.1	12.0 ± 1.4
Physical activity				
No	117 (42)	55 (35)	12.7 ± 1.5	12.1 ± 1.5
Yes	165 (58)	103 (65)	12.8 ± 1.6	12.8 ± 1.5
Alcohol use at 10–14 years				
No	271 (96)	153 (97)	12.8 ± 1.5	13.0 ± 1.5
Yes	11 (4)	5 (3)	11.8 ± 1.7	12.0 ± 2.5
Cigarette smoking at 10–14 years				
No	273 (97)	153 (97)	12.9 ± 1.6	12.7 ± 1.5
Yes	9 (3)	5 (3)	12.6 ± 1.4	12.2 ± 1.5

—, No observations.

**Table 2:** Frequency (%) of age of menarche and serum TCDD levels (ppt) of women who were premenarcheal at exposure, SWHS, Seveso, Italy, 1996–1998.

	All premenarcheal women in 1976		Women < 8 years in 1976	
Age at menarche (years)	No. (%)	Serum TCDD Median (IQR)*	No. (%)	Serum TCDD Median (IQR)**
< 11	16 (6)	183.5 (107–292)	13 (8)	167.0 (96–244)
11	36 (13)	109.7 (42–323)	22 (14)	231.5 (84–553)
12	83 (29)	122.0 (45–288)	48 (30)	179.0 (59–477)
13	56 (20)	207.5 (74–324)	32 (20)	221.5 (78–403)
14	62 (22)	135.0 (51–214)	27 (17)	176.0 (128–407)
> 14	29 (10)	136.0 (51–340)	16 (10)	163.0 (50–570)
Total	282 (100)	140.3 (56–300)	158 (100)	205.0 (76–417)

IQR interquartile range.

* ANOVA, *p* = 0.64.

** ANOVA, *p* = 0.97.

**Table 3:** Age at menarche (mean ± SD) of women who were premenarcheal at exposure and Cox proportional hazards estimates, SWHS, Seveso, Italy, 1996–1998.

	All premenarcheal women in 1976			Women < 8 years in 1976		
Exposure	No. (%)	Age at menarche (years)	HR (95% CI)	No. (%)	Age at menarche (years)	HR (95% CI)
Log_10_TCDD	282	12.8 ± 1.6	0.95 (0.83–1.09)	158	12.5 ± 1.5	1.08 (0.89–1.30)
TCDD (ppt)						
≤ 20	24 (8)	13.1 ± 1.7	1.11 (0.75–1.64)	8 (5)	13.0 ± 2.0	0.76 (0.40–1.44)
20.1–55.9	47 (17)	12.7 ± 1.4	1.10 (0.83–1.45)	21 (13)	12.7 ± 1.6	0.94 (0.61–1.43)
56.0–140.2	70 (25)	12.8 ± 1.4	1.14 (0.90–1.42)	27 (17)	12.2 ± 1.4	1.20 (0.87–1.64)
140.3–300.0	72 (26)	12.6 ± 1.7	1.07 (0.85–1.35)	50 (32)	12.4 ± 1.8	1.01 (0.75–1.35)
> 300.0	69 (24)	12.8 ± 1.6	1.00	50 (33)	12.7 ± 1.3	1.00

CI, confidence interval.
